# Dexmedetomidine as an Anesthetic Adjuvant in Cardiac Surgery: a Cohort Study

**DOI:** 10.5935/1678-9741.20160043

**Published:** 2016

**Authors:** Paulo Gabriel Melo Brandão, Francisco Ricardo Lobo, Serginando Laudenir Ramin, Yasser Sakr, Mauricio Nassau Machado, Suzana Margareth Lobo

**Affiliations:** 1 Division of Critical Care Medicine, Faculdade de Medicina de São José do Rio Preto (FAMERP), São José do Rio Preto, SP, Brazil.; 2 Division of Anesthesiology, Department of Surgery, Faculdade de Medicina de São José do Rio Preto (FAMERP), São José do Rio Preto, SP, Brazil.; 3 Department of Anesthesiology and Intensive Care Medicine, Friedrich-Schiller-University, Jena, Germany.; 4 Division of Cardiology. Faculdade de Medicina de São José do Rio Preto (FAMERP), São José do Rio Preto, SP, Brazil.

**Keywords:** Dexmedetomidine, Cardiovascular Surgical Procedures, Adjuvants, Anesthesia

## Abstract

**OBJECTIVE:**

α-_2_-agonists cause sympathetic inhibition combined with parasympathetic activation and have other properties that could be beneficial during cardiac anesthesia. We evaluated the effects of dexmedetomidine as an anesthetic adjuvant compared to a control group during cardiac surgery.

**METHODS:**

We performed a retrospective analysis of prospectively collected data from all adult patients (> 18 years old) undergoing cardiac surgery. Patients were divided into two groups, regarding the use of dexmedetomidine as an adjuvant intraoperatively (DEX group) and a control group who did not receive α-_2_-agonist (CON group).

**RESULTS:**

A total of 1302 patients who underwent cardiac surgery, either coronary artery bypass graft or valve surgery, were included; 796 in the DEX group and 506 in the CON group. Need for reoperation (2% *vs.* 2.8%, *P*=0.001), type 1 neurological injury (2% *vs.* 4.7%, *P*=0.005) and prolonged hospitalization (3.1% *vs.* 7.3%, *P*=0.001) were significantly less frequent in the DEX group than in the CON group. Thirty-day mortality rates were 3.4% in the DEX group and 9.7% in the CON group (*P*<0.001). Using multivariable Cox regression analysis with in hospital death as the dependent variable, dexmedetomidine was independently associated with a lower risk of 30-day mortality (odds ratio [OR]=0.39, 95% confidence interval [CI]: 0.24-0.65, *P*≤0.001). The Logistic EuroSCORE (OR=1.05, 95% CI: 1.02-1.10, *P*=0.004) and age (OR=1.03, 95% CI: 1.01-1.06, *P*=0.003) were independently associated with a higher risk of 30-day mortality.

**CONCLUSION:**

Dexmedetomidine used as an anesthetic adjuvant was associated with better outcomes in patients undergoing coronary artery bypass graft and valve surgery. Randomized prospective controlled trials are warranted to confirm our results.

**Table:** 

**Abbreviations, acronyms & symbols**	
CABG	= Coronary artery bypass graft
CI	= Confidence interval
CON	= Control
CPB	= Cardiopulmonary bypass
DEX	= Dexmedetomidine
IABP	= Intra-aortic balloon pump
ICU	= Intensive care unit
IQ	= Interquartile range
MAC	= Minimal alveolar concentration
OR	= Odds ratio
SD	= Standard deviation

## INTRODUCTION

Cardiac anesthesia has changed over the years from using high doses of opioids to fast-track surgery. High doses of opioids were justified based on the hemodynamic stability provided even in patients with marginal cardiac reserve^[1]^, but resulted in patients requiring prolonged mechanical ventilation support. Limited financial resources and a shortage of intensive care unit (ICU) beds have increased the need to optimize treatment and avoid complications in the postoperative period. Fasttrack cardiac anesthesia uses methods, such as short acting neuromuscular blockers, low-doses of opioids or regional anesthesia, with the intention of speeding up weaning from ventilation and decreasing ICU length of stay^[2]^. Fast-track cardiac anesthesia is being adopted more and more widely to reduce ICU bed use and hospital costs associated with postoperative care^[3-5]^. The safety and ability of this approach to diminish time on the ventilator have been demonstrated recently in a large cohort of almost 8000 patients^[5]^. Use of anesthetic adjuvants with opioids sparing qualities may create alternatives that accelerate weaning from mechanical ventilation and patients recovery.

Many beneficial effects of α-_2_-agonists have been reported, including a decrease in sympathetic tone with attenuation of hemodynamic and neuroendocrine responses to stress^[6]^ and reduced anesthetic and opioid requirements^[7]^. Dexmedetomidine has analgesic, sedative, anxiolytic and sympatholytic properties. Patients in the ICU sedated with dexmedetomidine alone wake when requested, become cooperative and the drug does not cause respiratory depression^[8]^. Herr et al.^[9]^ demonstrated the safety and benefits of using dexmedetomidine in comparison to propofol sedation in the postoperative period of coronary artery bypass graft (CABG) surgery. In a double-blind randomized study, Jalonen et al.^[10]^ compared dexmedetomidine to fentanyl as an anesthesia adjunct during CABG. They reported reduced sympathetic tone, attenuated hyperdynamic responses with dexmedetomidine and a reduction on additional fentanyl doses during the operation before bypass and postoperatively^[10]^.

The primary aim of our study was to evaluate the effects of dexmedetomidine as an anesthetic adjuvant during CABG and valve surgery on mortality and on the length of stay in the ICU compared to a control group receiving general balanced anesthesia.

## METHODS

This study was a retrospective analysis of an electronic database with data prospectively collected from January 2003 to April 2011. The data were retrieved from the databank collected for a previously published study registered in Clinical Trials.gov (NCT00780845) by one of the authors (MNM)^[11]^. Because of the retrospective nature of the study, the need for written informed consent was waived by the local ethics committee.

Either propofol (2 mg/kg) or etomidate (0.2 mg/kg) were used for anesthesia induction. Isoflurane was used in minimal alveolar concentration (MAC) range of 0.8-1.2 for anesthetic maintenance in both groups, depending on the anesthetic depth required. Neuromuscular block was achieved with an infusion of atracurium at 8 µg/kg/min. The anesthetic adjuvant was used at the discretion of the anesthesiologist, according to his/her experience. Patients were divided into two groups according to whether they had received dexmedetomidine (DEX group), given as a loading dose of 0.5 µg/kg over 20 minutes for anesthesia induction and a maintenance infusion of 0.5 µg/kg/h, or a control group (CON group) technique without anesthetic adjuvant. Both groups could receive doses of fentanyl as necessary (bolus of 1-5 µg/kg).

The preoperative estimation for risk of hospital death was calculated using the Logistic EuroSCORE^[12]^. Left ventricular ejection fraction was estimated for each patient using M-mode echocardiography based on the cubed or Teicholz method, wall motion scoring, or biplane Simpson's methods, or ventricular angiography. Data were collected on time on cardiopulmonary bypass (CPB), need for dialysis or intra-aortic balloon pump (IABP), mortality at 30 days and need for reintubation because of pulmonary complications. Acute kidney injury was assessed based on the AKIN classification^[13]^. Type 1 neurological injury was defined as a new episode of motor deficit, coma, seizure or encephalic lesion documented by cranial computed tomography or magnetic resonance imaging. Hospital mortality and hospital discharge dates were available for all patients from electronic hospital records.

Data were analyzed using SPSS Statistical version 17 and StatsDirect Software version 2.7.8. Descriptive statistics were computed for all study variables. The Kolmogorov-Smirnov test was used to verify the normality of distribution of continuous variables. Non-parametric tests of comparison were used for variables evaluated as not being normally distributed. Difference testing between groups was performed using Mann-Whitney U, Chi-square and Fisher's exact tests as appropriate.

A survival analysis was performed using Kaplan-Meier graphs at 30 days and the log rank test was used to compare the survival curves. We performed a multivariable logistic regression analysis with 30-day mortality as the dependent variable to evaluate the possible influence of dexmedetomidine on 30-day mortality after adjusting for possible confounders. In this analysis, we included age, gender, the logistic EuroSCORE, body mass index, dexmedetomidine, CABG or valve surgery, diabetes mellitus, moderate or severe left ventricular dysfunction, number of grafts, on-pump CABG, duration of CPB and use of IABP. Colinearity was excluded prior to modeling (R^2^>0.6 for all pairwise correlations). None of the included variables was colinear. A Hosmer and Lemeshow test was performed to assess the goodness of fit for the final model. In the case of categorical variables, a reference category was defined. Odds ratios (OR) and 95% confidence intervals were computed. Continuous variables are presented as mean ± standard deviation (SD) or median [25-75% interquartile range] (IQ) and categorical variables as number and percentage unless otherwise indicated. All statistics were two-tailed, and a *P*<0.05 was considered significant.

## RESULTS

We included 1302 consecutive patients who underwent CABG (n=817) or valve (n=485) surgery during the study period (63% male; age =54.7±13.2 years): 796 patients in the DEX group and 506 patients in the CON group. The characteristics of the study groups are shown in Table 1. The overall 30-day hospital mortality was 5.8%.

**Table 1:** Baseline characteristics.

	**CON group (n=506)**	**DEX group (n=796)**	***P*-value**
Male (%)	312 (62)	506 (64)	0.487
Age, years; median [IQ]	58 [47-67]	56 [47-64]	0.013
EuroSCORE (Additive)	3 (2-5)	3 (1-4)	<0.001
EuroSCORE (Logistic)	4.0±5.4	2.8±2.9	<0.001
Diabetes mellitus	110 (22)	174 (22)	0.959
Moderate/severe LVD	118 (23)	153 (19)	0.076
CABG n (%)	310 (61)	507 (64)	0.377
On-pump surgery n (%)	421 (83)	616 (77)	0.011
Number of grafts	3 (2-3)	2 (2-3)	0.352
Duration of CPB	94 [79–112]	88 [74-105]	<0.001
Valve surgery	196 (39)	289 (36)	0.377

Patients in the DEX group were younger (54.7±13.2 years *vs.* 56.5±13.1 years, *P*=0.013) and had a lower additive EuroSCORE (3; 1-4 *vs.* 3; 2-5, *P*<0.001) than patients in the CON group. More patients in the CON group (83%) underwent on-pump surgery than in the DEX group (77%) (*P*=0.011). Time on CPB was longer in the CON group (94 minutes) than in the DEX group (88 minutes) (*P*<0.001) (Table 1).

ICU length of stay was significantly shorter for patients in the DEX group (3.7±4.4 days) than for patients in the CON group (4.4±6.3 days) (*P*=0.02). Thirty-day hospital mortality rates were 3.4% in the DEX group and 9.7% in the CON group (*P*<0.001) (Table 2).

**Table 2:** Outcome parameters in the study groups.

	**CON group (n=506)**	**DEX group (n=796)**	***P*-value**
Reintubation	58 (11.0)	67 (8.4)	0.069
Need for reoperation	10 (2.0)	22 (2.8)	0.371
Atrial fibrillation	47 (9.3)	52 (6.5)	0.067
Acute kidney injury	135 (27.0)	190 (24.0)	0.253
Type 1 neurological injury	24 (4.7)	16 (2.0)	0.005
ICU readmission	28 (5.5)	28 (3.5)	0.081
ICU LOS (days); mean ± SD	4.4±6.3	3.7±4.4	0.020
30-day hospital mortality rates	49 (9.7)	27 (3.4)	<0.001

ICU=intensive care unit; LOS=length of stay; SD=standard deviation

**Fig. 1:**
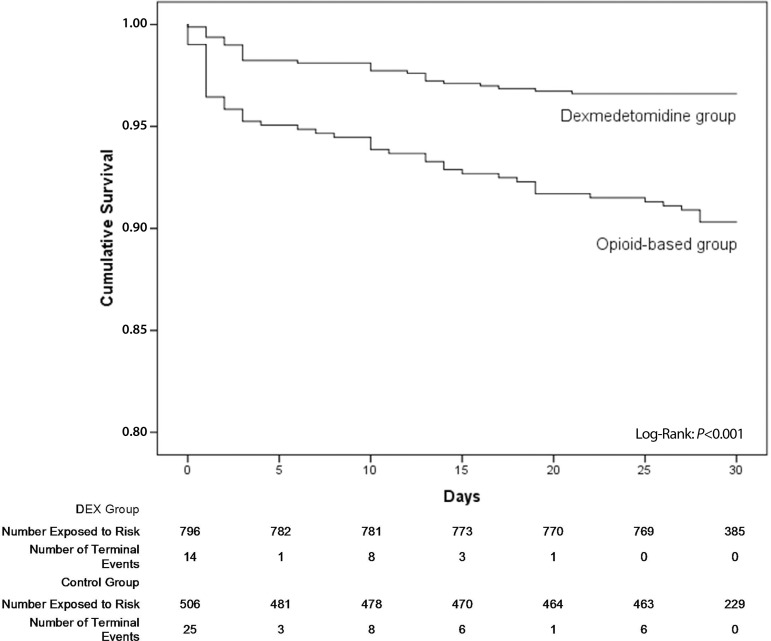
Kaplan-Meier survival curves.

The Kaplan-Meier survival curves for the DEX and CON groups are shown in Figure 1 (log-rank and Wilcoxon = *P*<0.001). Results from the multivariable logistic regression analysis with death as the dependent variable are shown in Table 3. Use of dexmedetomidine was independently associated with a lower risk of 30-day mortality OR=0.39, 95% confidence interval (CI): 0.24-0.65, *P*≤0.001). The Logistic EuroSCORE (OR=1.05, 95% CI: 1.02-1.10, *P*=0.004) and age (OR=1.03, 95% CI: 1.01-1.06, *P*=0.003) were independently associated with a higher risk of 30-day mortality.

**Table 3:** Multivariable logistic regression analysis with 30-day hospital mortality as the dependent variable.

	**Odds ratio CI (95%):**	***P*-value**
Dexmedetomidine	0.39 (0.24-0.65)	<0.0001
Logistic EuroSCORE	1.05 (1.02-1.10)	0.004
Age (per year)	1.03 (1.01-1.06)	0.003
Gender (Male *vs*. Female)	0.71 (0.43-1.18)	0.19
CABG *vs*. Valve	0.65 (0.36-1.17)	0.15
Diabetes mellitus	1.19 (0.66-2.16)	0.56
Left ventricular dysfunction	1.31 (0.74-2.3)	0.35

CABG=coronary artery bypass graft surgery; CI=confidence interval Hosmer and Lemeshow Chi square = 10.3 (*P*=0.244) Nagelkerke R-square = 0.109

## DISCUSSION

The main finding of this study in a large cohort of cardiac surgery patients is that use of dexmedetomidine, an α-_2_-agonist, as an anesthetic adjuvant was independently associated with a lower risk of 30-day in-hospital death compared to the CON group. In addition, patients receiving dexmedetomidine spent less time in the ICU and had a lower frequency of type 1 neurological lesions.

The reasons for the better outcomes are not known, but is likely to be related to the blunting of stress response, greater hemodynamic stability, sparing effect on oxygen consumption, protection of perioperative ischemia and preservation of neutrophil/ cellular immune function reported with the use of dexmedetomidine in comparison to opioids^[10,14,15]^.

Accordingly, Wijeysundera et al.^[16]^ performed a systematic review of studies evaluating the use of α-_2_-agonists (clonidine, dexmedetomidine or mivazerol) perioperatively for the prevention of cardiac complications among patients undergoing surgery. Overall, α-_2_-adrenergic agonists were associated with a more than 50% reduction in mortality in vascular surgery patients, in addition to reductions in cardiac mortality and myocardial infarction. Even when initiated after CBP infusion of dexmedetomidine was associated with decreased postoperative mortality up to 1 year and decreased incidence of postoperative complications and delirium in patients undergoing cardiac surgery^[17]^. In children undergoing complex congenital heart disease the use of dexmedetomidine after anesthesia induction was associated with less variability in heart rate and blood pressure than with the use of propofol^[18]^.

We found a decreased incidence of neurological problems in the DEX group in univariate analysis. The possibility of neuroprotection is speculative and should be tested prospectively. It has been suggested that α-_2_-adrenergic agonists improve neurological deficit scores and reduce infarct sizes in cerebral ischemia. These agonists improved the histomorphological and neurological outcomes after cerebral ischemic injury when administered during ischemia, and recent studies have provided considerable evidence that α-_2_-adrenergic agonists can protect the brain from ischemia/ reperfusion injury^[19]^. The mechanisms by which α-_2_-adrenergic agonists exert their neuroprotective effects are still unclear in humans since almost all studies regarding the neuroprotective effects of dexmedetomidine have been performed in animals^[20]^ or *in vitro*^[21]^. Sulemanji et al.^[22]^ demonstrated no benefit of dexmedetomidine as a neuroprotective agent during CABG surgery. Nevertheless, the use of dexmedetomidine as sedative agent in critically ill patients is associated with reduction in the incidence of delirium^[23]^. It is possible that the hemodynamic stability provided by dexmedetomidine may have favorable effects on the maintenance of cerebral perfusion^[10]^.

Older age and the CPB time were associated with a higher risk of death in our study. The increased morbidity and mortality seen in elderly patients after cardiac surgical procedures is due to biological aging processes, but also to greater disease severity, associated comorbidities and surgical urgency than in younger patients^[24,25]^. Widyastuti et al.^[26]^ identified older age as predictor of prolonged mechanical ventilation following cardiac surgery. Older patients also have twice the incidence of postoperative stroke^[24]^. Both, older age and CPB time are linked to neurological dysfunction because of the augmented risk for microvascular cerebral obstruction and decline neurocognitive function^[27]^.

The present study has some important limitations; particularly its retrospective nature with potential bias. The heterogeneity in the baseline status of the groups regarding age, EuroSCORE and CPB time, are all likely to be related to worse outcomes. The CON group had a higher mortality than it is expected on the literature. The EuroSCORE prognostic index did not reflect the mortality rate seen in this group, but it is not known if it is an applicable tool to our system of care. The agreement between the predict mortality by the EuroSCORE has been questioned by different centers in Brazil with conflicting results^[28,29]^, therefore a weight adjustment factor could probably correct its performance. In addition, the hospital where the study was performed is a reference in public health care system, being responsible to treat the most severe cases of a population that does not have access to primary health care. Indeed, the long observation period also carries a risk of other variables to be involved on the results. We corrected for a large number of possible confounders by performing a multivariate analysis, but it is possible that other, unknown, confounders were still present. Complications were analyzed at a univariate level; therefore a cause-effect relationship cannot be definitely established.

## CONCLUSION

Dexmedetomidine used as an anesthetic adjuvant was associated with better outcomes in patients undergoing CABG and valve surgery. Randomized prospective controlled trials are warranted to confirm our results.

**Table:** 

**Authors' roles & responsibilities**	
PGMB	Realization of operations and/or trials; final manuscript approval
FRL	Manuscript redaction or critical review of its content; final manuscript approval
SLR	Manuscript redaction or critical review of its content; final manuscript approval
YS	Statistical analysis; final manuscript approval
MNM	Conception and design study; analysis and/or data interpretation; manuscript redaction or critical review of its content; final manuscript approval
SML	Conception and design study; analysis and/or data interpretation; manuscript redaction or critical review of its content; final manuscript approval
